# Hospital Meetings, &c.

**Published:** 1901-05-04

**Authors:** 


### HOSPITAL MEETINGS, &c.

### EAST LONDON HOSPITAL FOR CHILDREN.

The festival dinner of the East London Hospital for Children was held at Grocers' Hall on April 30th, the Lord Mayor (Mr. Alderman Green) presiding.

In proposing the customary loyal toasts the Chairman announced that intelligence had that day reached the committee that His Majesty the King had been graciously pleased to become patron of the hospital—a statement that was received with cheers.

The Lord Mayor then gave the toast of the evening—"Success to the East London Hospital for Children." The very name, he said, was sufficient indication of how important the institution was. The public was never more ready to admit the claims of any branch of humanity than those of the children; and when they saw the teeming thousands of children in the East End and the amount of sickness prevailing among them, and how much they needed succour and relief in the hour of sickness, it was plain that if that hospital afforded a portion of that relief it was worthy of all support. The design of the institution was to maintain a hospital for children as in-patients, and a dispensary for women and children as out-patients, and also a convalescent home at the seaside, and for the work it was doing it deserved the warmest encouragement and support. Since its foundation in 1868 an average of 1,500 children had been admitted every year, 320 being subsequently sent to the seaside branch to complete their recovery; and every year over 35,000 were treated in the out-patient and casualty departments. It was thus no trifling or insignificant work this hospital was instrumental in carrying out. The annual cost was about £8,500, and that of the sea-side branch at Bognor £900—together £9,400. Towards this their only assured income was £875 from investments. The annual subscriptions totalled £2,200, leaving some £6,000 every year. For this they had to appeal to the generosity of the benevolent public, and beyond this they required something like £20,000 for additions. They wanted a new mortuary—towards the cost of which the Prince of Wales's Fund had contributed £500—a laundry, new kitchens, and improved accommodation for their nursing staff. They had to deplore the serious falling-off in the amount received from legacies—last year's was the smallest for ten years. He appealed with confidence for support, for the work was one which ought to prosper.

Mr. Charles Cheston, the treasurer, responded. The hospital's only fault, he claimed, was that it was out of sight. Half the citizens of London did not know there was such a place as Shadwell. He believed their work was the best done by any hospital in London—not that it was a better hospital, but that the population of its district had the strongest claims on the charity of the people at large. He mentioned that a Ladies' Association was being formed, and said he had heard it suggested that next year the dinner should be presided over by a lady; that the guests should be ladies; and that men should only be admitted on presentation of a cheque duly filled in and signed. (Laughter.)

The Hon. Alban Gibbs, M.P., proposed "The Board of, Management and the Medical Staff," to which Lord Falkland responded.

It was announced that the amount collected was £1,873.

### METROPOLITAN HOSPITAL.

The sixty-fifth annual meeting of Governors of the Metropolitan Hospital, Kingsland Road, was held on Monday April 29th, Mr. Chas. J. Thomas, the chairman, presiding.

The report presented gave the receipts for 1900 as £12,431, the largest amount ever received in one year—a result especially gratifying in view of the various war funds. The grant of the Prince of Wales's Fund had been increased from £500 in 1898 and £1,000 in 1899 to £1,500, this handsome donation being made after a thorough inspection of the institution. Donations had increased from £3,278 to £5,634, but legacies had decreased from £5,118 in 1898 and £2,703 in 1899 to only £1,405 in 1900. The ordinary expenditure was £10,987, and the extraordinary expenditure (repairs and building improvements) £228. There were 964 in-patients as compared with 867 in 1899, and 33,708 out-patients, with 106,898 attendances, as against 31,245, with 101,597 attendances. The committee were again able to place 36 beds (in two empty wards) at the disposal of the Metropolitan Asylums Board for enteric fever cases, the board providing the necessary beds and bedding and agreeing to pay a sum to cover the cost to the hospital. In consequence of this the number of beds available was, during seven months, 76; and during five months, 112, the daily average occupied being 79½. The average stay of each in-patient was 30 days. Regret was expressed at the death of Mr. Coleman Defries, who had been actively associated with the hospital for nearly 40 years, of Dr. Sedgwick Saunders, and of Dr. Dudley, one of the honorary consulting physicians, who first found the staff in 1867. Mr. Frederick Snowden and Mr. Frederic Franklin had been elected trustees to the hospital to fill the vacancies caused by the death of Mr. Defries and of Mr. Joseph Fry. Sisters Thorpe and Tate, who volunteered for South Africa, were still at Pretoria with No. 2 General Hospital. The Ladies' Association, founded in 1899, had proved a great success, and had now a membership of over 150. A concert given under its auspices in May last at the Queen's Hall realised £475. The festival dinner last year resulted in a record amount, £4,444, being received. The Cranbrook Convalescent Home had continued to be of the greatest benefit to the hospital. The committee expressed their sense of encouragement at the improved financial position of the hospital. They desired strongly to be provided with increased funds to enable them to open 26 more beds, and so bring up the number always available to 100, and recorded that towards this extra expenditure the trustees of the late Mr. Zuntz had allocated to the Metropolitan Hospital £1,000 a year for 10 years from the funds left at their discretion for assisting hospital work in the Metropolis. The report concluded with the announcement that the King had consented to continue his patronage of the hospital.

The Chairman, in moving the adoption of the report, referred to the various points mentioned in it, and said the committee had decided to open the extra beds contemplated in July. They would not, however, be satisfied till they were able to open the 160 beds for which the hospital was designed.

The report was adopted, the committee was re-elected, and votes of thanks concluded the proceedings.

### LONDON LOCK HOSPITAL.

Lord Kinnaird, Chairman of the Board of Management, presided at the 154th annual meeting of the London Lock Hospital on Thursday, April 25th. The report presented referred to the death of Queen Victoria, and mentioned that the King, who, as Prince of Wales, held the office of patron for twenty-seven years, had consented to continue his patronage. The year 1900 commenced with a debt of £2,314, and this had unfortunately increased by the end of December to £3,582. The total ordinary receipts amounted to £8,441, and the total ordinary expenditure to £9,424. The extraordinary expenditure was £230—£160 of which was for a new fumigating machine. The Prince of Wales's Fund had contributed a special grant of £500. The number of male in-patients admitted was 253, and of female in-patients 627. There had been some difficulty in finding room for all the latter class that had applied for admission, but the number received was smaller than in previous years, it having been decided to keep cases longer than previously. The male out-patients numbered 13,870, and the female out-patients 2,120. Some important additions had been made in the two hospitals and home, and much of the existing plant had been renewed. In the female hospital a Washington Lyons steam disinfector had been erected. The working of this would involve little expenditure, the steam being already there. Prior to its erection the hospital and home were without any proper means of bringing clothes and bedding into a satisfactory state. The wards of the male hospital had been installed with the electric light. One of the empty wards had just been opened and the other handed over to the home as a temporary arrangement. During the year 90 patients passed into the home, being considerably more than in any previous year, while several were placed in other homes, 35 were sent to service, 22 restored to friends, 18 sent to other homes, while 2 left at their own request. The employments given to the inmates of the home were laundry and household work, to which had been added the making of stockings by machine. The amount earned by laundry work was £732, being slightly in excess of the previous year.

The chairman moved the adoption of the report, which was carried, and votes of thanks brought the proceedings to a close.

